# Direct-Acting Antivirals in Kidney Transplant Patients: Successful Hepatitis C Treatment and Short-Term Reduction in Urinary Protein/Creatinine Ratios

**DOI:** 10.20411/pai.v2i3.211

**Published:** 2017-09-19

**Authors:** Michael R. Goetsch, Ashutosh Tamhane, Mohit Varshney, Anuj Kapil, Edgar T. Overton, Graham C. Towns, Ricardo A. Franco

**Affiliations:** 1 School of Medicine, University of Alabama at Birmingham, Birmingham, Alabama; 2 Department of Medicine, Division of Infectious Diseases, University of Alabama School of Medicine, Birmingham, Alabama; 3 Department of Medicine, Division of Nephrology, University of Alabama School of Medicine, Birmingham, Alabama

**Keywords:** direct-acting antivirals, kidney transplant, hepatitis C, protein creatinine ratios

## Abstract

**Background:**

The role of Hepatitis C Virus (HCV) clearance in kidney graft survival is unknown. We examined short-term trends of protein/creatinine (P/C) ratios in HCV-infected kidney transplant recipients treated with direct-acting antivirals (DAAs).

**Methods:**

This is a retrospective study of 19 kidney transplant patients with HCV infection treated with DAAs at the University of Alabama at Birmingham between January 2013 and June 2016. Markers of glomerular damage were assessed using average urinary protein/creatinine (P/C) ratios measured pretreatment and posttreatment. Treatment efficacy was defined as sustained virologic response at 12 weeks post-HCV treatment (SVR12).

**Results:**

The median age of the 19 patients included was 59 years (Q1 = 58, Q3 = 64). Of these patients, 68% were African American, 32% were White and 63% were male. The median time between kidney transplant and initiation of DAA therapy was 2.25 years (Q1 = 0.79, Q3 = 3.79). Posttreatment P/C ratios (median = 0.127, Q1=0.090, Q3 = 0.220) were significantly lower (*P* = 0.01) than pretreatment ratios (median = 0.168, Q1 = 0.118, Q3 = 0.385). P/C ratios decreased in 14 of 19 patients (74%) with a median change of -0.072 (median percent change = -40%). Post-treatment estimated glomerular filtration rates (median = 58.9, Q1 = 48.9, Q3 = 72.3) were not significantly different (*P* = 0.82) than the pretreatment values (median = 57.0, Q1 = 48.8, Q3 = 67.8). All patients achieved SVR12.

**Conclusions:**

In this preliminary study, there was a statistically significant decrease in P/C ratios associated with HCV clearance, suggesting a potential role for DAAs in improving kidney graft survival. Larger cohort studies will be needed to assess the clinical and long-term benefits of DAAs in this population.

## INTRODUCTION

More than 170 million people worldwide are infected with hepatitis C virus (HCV)—a leading cause of liver disease morbidity and mortality [1]. HCV is considered a risk factor for chronic kidney disease (CKD) progression, posttransplantation glomerular disease, rejection and poorer kidney graft survival [2–4]. Interferon and ribavirin-based therapy use is limited in the transplant setting due to bone marrow suppression, renal dysfunction, and kidney-graft rejection [5, 6].

The advent of direct-acting antivirals (DAAs) brings greater efficacy, fewer adverse effects, and holds great potential in addressing unmet needs of HCV-positive kidney transplant recipients [7]. The evidence for safety and efficacy in this population is evolving with promising results [8–12]. However, the impact of DAA use in improving markers of kidney-graft damage—such as the urinary protein/creatinine (P/C) ratio—remains unclear. Because increasing numbers of kidney transplant recipients are expected to undergo HCV treatment in the near future, it is critical to examine the effects of virus eradication on markers of graft glomerulopathy that are routinely monitored in the clinic. This will be instrumental in understanding the impact of HCV treatment on long-term kidney graft survival.

In this study, we examined the short-term trends of urinary P/C ratios in a cohort of HCV-infected kidney transplant recipients treated with DAAs. We also report our own experience with DAA safety and efficacy in this population.

## METHODS

We conducted an observational retrospective study of kidney transplant recipients treated for HCV infection with DAAs at the University of Alabama at Birmingham (UAB) 1917 Viral Hepatitis Clinic and the UAB Liver Center from January 2014 through August 2016. We screened charts and collected data using the UAB electronic medical record system (EHR) (Cerner, Kansas City, MO). This study was approved by the UAB institutional review board (protocol No. X150312011).

All included participants (N = 19) had HCV infection confirmed with genotype identification on first referral to the viral hepatitis clinic, prior to initiation of DAA therapy. The DAA combination was selected by the treating physicians on the basis of best practices available at the time of treatment (www.hcvguidelines.org). All patients had an estimated glomerular filtration rate (eGFR) > 30 mL/minute before initiating therapy. We collected information for age, sex, race, type of allograft donor, HCV genotype, history of liver transplant, cirrhosis status, prior HCV treatment, DAA regimen, HCV viral load at baseline, sustained virologic response at 12 weeks posttreatment (SVR12), reported treatment side effects and adverse events, pretreatment and posttreatment urinary P/C ratios, pretreatment and posttreatment eGFRs, immunosuppression regimens, and hepatitis B virus (HBV) and human immunodeficiency virus (HIV) serostatus. Liver fibrosis staging was evaluated by means of liver biopsy, ultrasound and/or transient elastography (TE). One patient had ultrasound only. All others had both ultrasound and TE, and 2 patients had liver biopsy. Cirrhosis was defined as liver biopsy results showing a Metavir fibrosis score of 4, a FibroScan (Echosens) read greater than 12.5 kPa, or by ultrasound imaging reporting the nodular contour of the liver or other stigmata of portal hypertension.

Markers of glomerular damage were assessed using serial urinary P/C ratios obtained through routine post-kidney transplant clinical care. Guidelines from KDIGO (Kidney Disease: Improving Global Outcomes) recommend quarterly urinary proteinuria screening during the first year post-transplant, followed by annual screening [6]. At our institution, laboratory tests are performed monthly for the first 3 years posttransplant, and quarterly thereafter. For each participant, we extracted the 3 longitudinal P/C ratios immediately preceding and following HCV treatment and calculated mean P/C ratios before and after DAA therapy. Trends in kidney graft function were evaluated similarly using mean eGFR pretreatment and posttreatment. The values for eGFR were calculated using the Modification of Diet in Renal Disease Study equation (MDRD) as well as the Chronic Kidney Disease Epidemiology Collaboration equation (CKD-EPI). We assessed treatment efficacy using SVR12. We assessed DAA safety in this population by collecting data on side effects and adverse events reported in the EHR.

## STATISTICAL ANALYSIS

Initial data evaluation began with descriptive statistics. Continuous variables (eg, P/C ratio, eGFR) were expressed as medians with quartiles (Q1, Q3) as the distribution was skewed. Categorical variables (eg, SVR12) were expressed as frequencies with percentages (%). The statistical significance of the difference for the P/C ratio and eGFR, both being measured before and after the treatment, was examined using the Wilcoxon signed-rank test. Statistical significance was set at 0.05 (2-tailed). Analysis was conducted using SAS software, version 9.3 (SAS Institute Inc., Cary, NC).

## RESULTS

Nineteen kidney recipients completed HCV treatment with DAAs during the study period. Table 1 lists the distribution of the HCV treatment and immunosuppressive regimens used, donor status, and other key clinical characteristics. The median age was 59 years (Q1 = 58, Q3 = 64) at completion of treatment. Of these patients, 68% were African American, 32% were White, and 63% were male. Four of 19 patients (21%) were positive for HBV core antibody, indicating past HBV infection, although none of the patients had positive HBsAg at the initiation of DAA therapy. Seven patients had liver fibrosis staging consistent with cirrhosis by at least 1 staging modality. The 2 patients who received liver transplants did so 15 and 5 years prior to receiving kidney transplants. Ten patients were prescribed Angiotensin-Converting-Enzyme Inhibitors (ACEi) or Angiotensin II Receptor Blockers (ARB). Of these, 9 patients were prescribed ACEi or ARB at least 1 year prior to starting DAA therapy. One patient began ACEi treatment during the DAA use window, and there were no ACEi or ARB discontinuations during HCV treatment.

**Table 1.** Characteristics of the Kidney Transplant Recipients Treated for HCV Infection at the University Of Alabama at Birmingham (Birmingham, Alabama), January 2014–August 2016.

Subject	Race	Donor Status	HCV Gen	LT	Prior Rx	F4	DAA	ISS	eGFR	ACEi or ARB	HIV Status
1	AA	DD	1b	No	PR	No	LDV/SOF	TMP	69.1	Yes	Neg
2	W	DD	1b	No	No	No	LDV/SOF	CAP	46.9	Yes	-
3	W	LRD	1b	No	No	No	LDV/SOF	TMP	46.7	No	Neg
4	AA	DD	1a	No	No	No	LDV/SOF	TMP	97.2	No	Neg
5	AA	DD	1a	No	No	No	LDV/SOF	CMP	52.0	Yes	Neg
6	W	LURD	1a	Yes	No	No	LDV/SOF	CMPS	48.0	Yes	Neg
7	AA	DD	1b	No	IR	No	SIM/SOF	TMP	80.5	No	Neg
8	AA	DD	1a	No	No	Yes	LDV/SOF	TMP	60.6	No	Neg
9	AA	DD	1b	No	PR	No	LDV/SOF	TM	57.0	Yes	Neg
10	W	DD	1a	No	No	Yes	LDV/SOF	TMP	27.9	No	Neg
11	AA	DD	1a	No	No	Yes	LDV/SOF	TMP	50.1	Yes	Neg
12	AA	DD	1b	No	No	No	LDV/SOF	TMP	65.2	Yes	Neg
13	W	DD	2	No	No	Yes	SOF/R	TMP	66.5	No	Neg
14	AA	DD	1a	No	No	No	LDV/SOF	TMP	61.2	No	Neg
15	W	DD	1b	Yes	IR	Yes	SIM/SOF	TMP	83.9	Yes	Neg
16	AA	DD	1a	No	No	Yes	LDV/SOF	TMP	80.2	No	Neg
17	AA	DD	1a	No	No	No	LDV/SOF	TMP	49.5	No	Neg
18	AA	LRD	1a	No	I	Yes	LDV/SOF	TM	50.0	Yes	Neg
19	AA	DD	1a	No	PR	No	LDV/SOF	TMP	45.3	Yes	Neg

Note: ‘-’ indicates patient received transplant in 1990. Paper archives and record of HIV test could not be located.

AA, African American; W, White. DD, deceased donor; LRD, living related donor; LURD, living unrelated donor. HCV, hepatitis C virus; Gen, Genotype. LT, Liver Transplant. Rx, Treatment; PR, pegylated interferon + ribavirin; IR, interferon + ribavirin; I, interferon. F4, liver fibrosis stage 4 (METAVIR). DAA, direct-acting antiviral regimen; LDV/SOF, sofosbuvir/ledipasvir; SIM/SOF, sofosbuvir/simeprevir; SOF/R, sofosbuvir/ribavirin. ISS, Immunosuppression; T, tacrolimus; M, mycophenolate mofetil; P, prednisone; C, cyclosporine; A, azathioprine; S, sirolimus. eGFR, estimated glomerular fltration rate. ACEi, Ace Inhibitor; ARB, angiotensin II receptor blocker. Neg, Negative.

The median time between kidney transplant and initiation of DAA therapy was 2.25 years (Q1 = 0.79, Q3 = 3.79) and the median viral load at the time of treatment initiation was 2,355,000 IU/ml (Q1 = 589,383; Q3 = 3,925,842). We observed a median time of 13 weeks between the pretreatment P/C ratio measurements midpoint and HCV treatment initiation. Likewise, the median time between the end of HCV therapy and the posttreatment P/C ratio measurements midpoint was 25 weeks. All patients were intended to receive 12 weeks of DAA therapy. However, one patient had a 10-day treatment interruption related to delays in insurance appeals. A patient with genotype 2 was treated with sofosbuvir/ribavirin for a total of 20 weeks because of a 3-week treatment interruption due to cryptococcal meningitis and later restarting therapy. All patients completed therapy and achieved SVR12. Reported side effects included headaches (16%), fatigue (16%), insomnia (5%), and rash (5%), although 47% of patients reported no side effects and 21% reported feeling better or having more energy post-HCV treatment.

**Figure 1.**
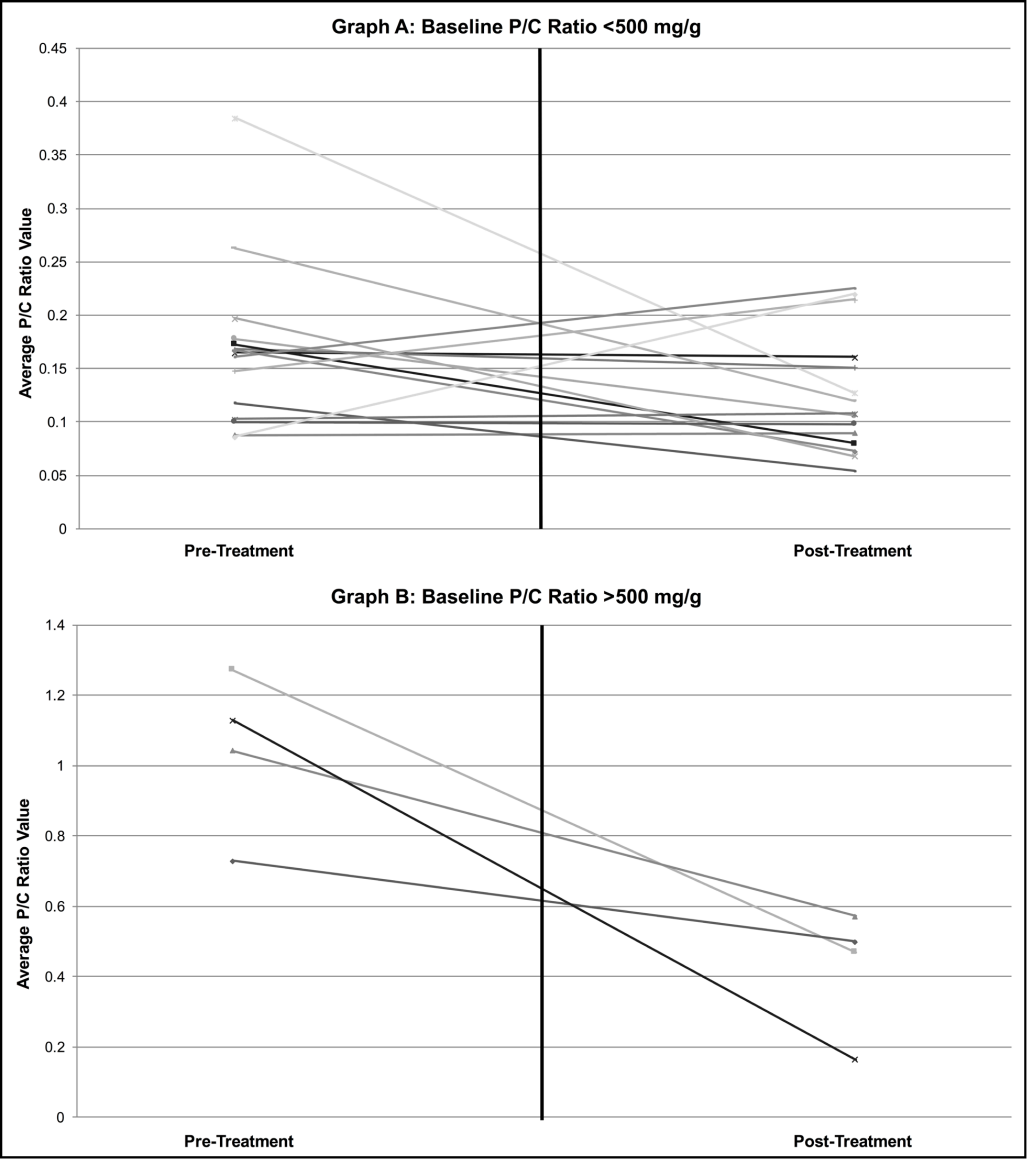
Trends in average P/C ratios pretreatment and post-HCV treatment for patients who had A) baseline average P/C ratios < 500 mg/g; and B) baseline average P/C ratios > 500mg/g. For each participant, average P/C ratios were extracted from 3 longitudinal P/C ratios obtained immediately preceding and following HCV treatment during routine care. P/C ratios decreased in 14 of 19 patients (74%) with median change of -0.072 (median percent change = -40%). Wilcoxon signed-rank test Overall, posttreatment P/C ratios (median = 0.127) were significantly lower (*P* = 0.01) than pretreatment ratios (median = 0.168).

Posttreatment P/C ratios (median = 0.127, Q1 = 0.090, Q3 = 0.220) were significantly lower (*P* = 0.01) than pretreatment ratios (median = 0.168, Q1 = 0.118, Q3 = 0.385). P/C ratios decreased in 14 of 19 patients (74%) with a median change of -0.072 (median percent change = -40%), as demonstrated in Figure 1. Posttreatment eGFRs (median = 58.9, Q1 = 48.9, Q3 = 72.3) were not significantly different (*P* = 0.82) than the pretreatment values (median = 57.0, Q1 = 48.8, Q3 = 67.8) when calculated with the MDRD formula. Analysis of eGFRs calculated with the CKD-EPI equation likewise showed no significant change with treatment (*P* = 0.90). One patient experienced an episode of antibody-mediated rejection diagnosed 12 months after completion of HCV treatment, related to low tacrolimus levels. The dose of tacrolimus was increased soon after HCV treatment with close follow-up with transplant nephrology staff. The patient had adequate social support, adherence to medicines and follow-up appointments, evidence of F3 liver fibrosis by non-invasive staging, and preserved clinical and laboratory liver function.

## DISCUSSION

We examined the short-term trends in mean P/C ratios in 19 kidney transplant recipients who underwent HCV treatment with DAAs. We observed statistically significant decreases in post-treatment mean P/C ratios relative to pretreatment. There was no change in eGFR, although we suspect that any effect of viral clearance on eGFR would only be manifested in the intermediate or long-term. All patients achieved SVR12, and the safety and efficacy profile was excellent. There were no serious treatment-related adverse events and side effects were minor and infrequent as described by the patients' self-reports. Taken together, these findings have important implications for future planning of larger, prospective studies examining the impact of HCV treatment in preventing proteinuria and posttransplantation glomerulonephritis, and promoting overall kidney graft survival.

Most patients in the study saw a decrease in proteinuria; the largest decreases were seen in patients whose pretreatment urinary P/C ratios exceeded 500 mg/g. This finding is in contrast to other recently-published studies. Gentil *et al* found no change in proteinuria in their cohort following HCV treatment. Compared to ours, their cohort had a longer median follow-up time post-kidney transplant (11.4 versus 2.25 years), implying longer exposures to other factors that can potentially lead to proteinuria and graft pathology such as calcineurin inhibitors, hypertension, and diabetes [13]. Lubetzky *et al* found that proteinuria increased post-HCV treatment in 6 (20%) patients [12]. Increases were seen in patients with baseline proteinuria ranging from 510 to 5470 mg/g. These patients were 65 years of age or older, and 4 had kidney biopsies (1 patient showing acute tubular injury, 1 with diabetic nephropathy, and the remaining 2 had signs of podocyte injury of unclear etiology). Given the small sample size and unmatched clinical characteristics of patients included in these study cohorts, further research is warranted to investigate the effects of DAA use in kidney transplant patients, especially within the subgroup with graft proteinuria > 500mg/g.

The decrease in proteinuria we observed was statistically significant, although the clinical implications for graft outcomes in the short or long term are unclear. Nevertheless, numerous studies have confirmed the association between proteinuria and poorer graft outcomes, even with non-nephrotic range proteinuria. Amer and Cosio have found that even low levels of urinary protein can portend significant graft pathology. They stratified hazard ratios according to protein-uria level and found 150 mg/24 hours (the cutoff between mild and moderate proteinuria) to be the threshold at which proteinuria begins adversely affecting graft survival [14, 15]. The average decreases with DAA therapy seen in our study were enough to reclassify patients from the moderate proteinuria ranges to mild. As reported by Amer and Cosio, such a decrease would be enough to potentially shift the hazard ratio from 4.1 to the null value in the long term [14].

The efficacy and safety profile was consistent with recent series. Sawinski and colleagues described outcomes in 20 patients who were predominantly treated for 12 weeks with sofosbuvir-based therapies for HCV genotype 1 infection [8]. SVR12 was achieved in 100% of patients with no early treatment discontinuations. Lin *et al* observed 24 patients who received sofosbuvir-based therapy with a SVR12 rate of 91% (21 of 23) [9]. One patient died prior to the SVR12 checkpoint due to a treatment-unrelated cause. Adverse events were reported in 11 patients (46%) and were managed without discontinuation of therapy. Fernández and colleagues verified a 98% achievement of SVR12 in 103 patients, 41% of whom received ribavirin-containing regimens. Anemia was observed in one-third of patients without treatment discontinuations [10].

In our study, 1 patient had verified antibody-mediated rejection diagnosed 12 months after completion of HCV treatment, and the rejection was attributed to low levels of tacrolimus despite a dose increase and close patient monitoring. Immunosuppression drug levels are monitored and adjusted according to our institution's standards of posttransplant routine care. However, the longitudinal and consistent analysis of calcineurin inhibitor (CNI) levels was not feasible due to changes in the drug level assay during our study period. In a study by Sawinski *et al*, 6 of 9 patients had verified decreases in CNI levels, and the authors pointed out the likely need for extended monitoring posttreatment [8]. Lin *et al* found that 2 of 16 patients with available trends for tacrolimus levels had significant decreases in levels during HCV therapy [9]. In the larger case series from Fernández *et al*, tacrolimus dosing needed adjustment in 62% of patients, more often by increased titrations, and 3 episodes of acute rejection were observed [10]. Several variables may affect CNI levels, including drug-drug interactions and possible enhanced metabolism of tacrolimus, especially when liver function of cirrhotic patients improves following SVR12 [8–10]. In light of the above, close and extended monitoring of CNI levels is warranted.

The results of our study should be interpreted considering its limitations. The study was small, retrospective, and had a short follow-up. Due to small sample size and multiple confounding factors that potentially influence glomerular pathogenesis in this population (diabetes, hypertension, use of ACEi and ARBs, fluctuations in immunosuppression levels, and others), this analysis cannot demonstrate causality between SVR12 and decreased mean P/C ratios. Furthermore, P/C ratios and reported side effects were obtained from routine care reports, unlike the standardized and systematic data collection of prospective studies. Nevertheless, levels of proteinuria have clearly been associated with worsening graft survival, and the use of P/C ratios in the transplant population bodes well for longitudinal assessment of graft injury in routine care. However, these measures can be influenced by several factors not controlled for in this study such as the source of proteinuria (although we suspect most treated patients did not have residual urine output in native kidneys), the etiology of proteinuria, and exercise and activity levels prior to urine collection [16].
